# Bird-spiders (Arachnida, Mygalomorphae) as perceived by the inhabitants of the village of Pedra Branca, Bahia State, Brazil

**DOI:** 10.1186/1746-4269-2-50

**Published:** 2006-11-13

**Authors:** Eraldo M Costa Neto

**Affiliations:** 1 Universidade Estadual de Feira de Santana, Department of Biology, Km 03, BR 116, 44031-460, Feira de Santana – Bahia, Brazil

## Abstract

This paper deals with the conceptions, knowledge and attitudes of the inhabitants of the county of Pedra Branca, Bahia State, on mygalomorph spiders locally known as 'caranguejeiras' (bird-spiders). It is launched here a new filed within ethnozoology: ethnoarachnology, which is defined as the transdisciplinary study of the relationships between human beings and bird-spiders. Data were collected from February to June 2005 by means of open-ended interviews carried out with 30 individuals, which ages ranged from 13 to 86 years old. It was recorded some traditional knowledge regarding the following items: taxonomy, biology, habitat, ecology, seasonality, and behavior. Results show that bird-spiders are classified as "insects". The most commented aspect of the interaction between bird-spiders and inhabitants of Pedra Branca is related to their dangerousness, since they said these spiders are very venomous and can cause health problems. In general, the traditional zoological knowledge of Pedra Branca's inhabitants concerning these spiders is coherent with the academic knowledge.

## Preamble

Bird-spiders belong to the Mygalomorphae suborder, Orthognatha group. Differently from the spiders of the Araneomorphae suborder, in this group of spiders the chelicerae move in parallel with the longitudinal axis of the body, there are two pairs of lungs, and their venom glands are situated entirely inside the basal segment of the chelicerae [1]. There are nearly 2.547 known species and they are found in all continents, except Antarctica (Paulo César Motta, personal communication, 2006). Considering the family Theraphosidae, a total of 897 and 170 species have already been described in the world and in Brazil, respectively [2]. They grow up through skin changes, and females still go through these changes even after they are adults [3]. *Theraphosa blondi *(Latreille, 1804), which lives in the Amazon forest, is up to 26 cm long [4].

These spiders usually dig burrows when they are spiderlings and live in them for many years, enlarging them as necessary. Once established at a site, an individual tarantula usually spends its life there, hunting in an area only a few meters adjacent to its burrow [5]. According to Smith [5], courtship involves cautious approach by the male, who touches the female with his front legs, then moves in a way that identifies both his gender and species. If the female is receptive to his tentative advances, she abandons her burrow and ultimately postures with her cephalothorax raised. She remains inactive while the male approaches her from the front. He uses special hooks on the first pair of his walking legs to hold the female's fang-bearing chelicerae during insemination. After the sperm have been deposited in the female's abdominal genital opening, the male disengages his hooks and departs briskly.

Erroneously, mygalomorphs are referred to as tarantulas by many people, but the true tarantulas are araneomorph spiders of the widespread genus *Lycosa *[6]. The name 'tarantula' comes from a cult in Taranto, Southern Italy where the bite of a spider (*Lycosa *and *Latrodectus*) served as a pretext for dionisiac reunions of a frenetic dance [7]. The symptoms of the affliction may include dizziness, weakness, feelings of anguish, psychomotor agitations, stomach and muscular pains, nausea and increased erotic appetite in its acute phase [8]. This author states that tarantism is related to Saint Paul's legend of the eradication of venomous creatures from Malta. In common sense, however, the term tarantula means almost all big and hairy spiders.

On the other hand, the name 'bird-spider' comes from the fact that some mygalomorphs feed onsmall birds. The first European explorers in South America named the spider after seeing a specimen (*Avicularia avicularia *Linnaeus, 1758) eatinga small bird. Another reason touse this name could be that some tree spiders drop to the ground when there is danger, spreading their legs that are covered with hair, and gliding like a little bird from branch to branch. In Central America they are called gorse-spiders because of the mistaken idea that their bite can cause a horse's hoof to fall off [7]. In Colombia giant bird-spiders of the genus *Theraphosa *are named *rebienta*-*caballo *or *mata*-*caballo *(killer horse) [9].

These 'big spiders' are culturally significant for different human societies throughout the world [10]. They can be found in myths and legends, and usually play important roles in daily life of many cultures [11]. In Cameroon, for example, traditional seers use them to foretell the future: the seer sets a container over the spider's burrow, and places an assortment of elaborately patterned leaf cards with advices around the entrance. The spider has to rearrange the cards to yield such advices [12]. In Laos people are used to eating bird-spiders. They take their fangs off, then they roast them in broaches, and after that they eat them with salt or in pepper sauce. Sometimes, however, parts of these spiders, usually abdominal contents or the eggs of the big females, are eaten raw [13]. The Khmer of Cambodia are reported to eat large theraphosids deep-fried in oil and served on skewers [14]. The Piaroa and the Yanomamo Indians of the Amazon Basin eat roasted *Theraphosa blondi *and other large species, which they extract from their burrows [14,15]. Paoletti and Dufour [16] have summarized all available data for Amazon.

The medicinal use of bird-spiders has also been recorded. Among the Tzeltal from Chiapas, Mexico these arthropods are used for treating tumors. They are induced to sting the affected area [17]. In Brazil the 'tooth' of a bird-spider is attached around the neck in order to treat erysipelas [18], while its fangs are recommended against toothache in the state of Alagoas [19]. These 'teeth' are also used as an amulet that is attached on a child's neck to rid it of evil eyes [20]. Cascudo [21] recorded the usual belief that a child who wears such amulets will have a white, strong, resistant denture. In the city of Feira de Santana, Bahia State, the powder of a toasted bird-spider is turned into a tea and offered for whom suffer from asthma [22].

Although many people consider bird-eater spiders as repulsive and dangerous creatures (helped by horror movies such as *Tarantula*), there is an increasing proportion of the world's population that actually enjoys the company of these arachnids, since a number of them are now popular as household pets. Unfortunately a great amount of specimens comes from dealers who travel to tropical countries searching for these large spiders [23]. This has endangered a few species, such as the Mexican red-kneed *Brachypelma smithi *(Cambridge, 1897) which has been over-collected and is now cited in the CITES Appendix II [6].

Despite the close association between spiders and human communities, few ethnobiologists and anthropologists have paid little attention to knowledge and use of them [24]. Considering that ethnozoological studies on bird-spiders are rare in Brazil [25,26], this paper deals with the perception, knowledge and behavior of these spiders in a small community from Bahia State, Northeastern Brazil. It is a brief contribution towards a new branch of research, ethnoarachnology.

## Materials and methods

Fieldwork was carried out in the village of Pedra Branca, which is located in the East region of Bahia State, northeastern Brazil. This settlement belongs to the municipality of Santa Terezinha, and it is about 13 km away from it. It is situated at the base of the Serra da Jibóia, a mountain range of about 225 km^2 ^of area whose peak elevation is 805 m above sea level. It lies between 12°46' South and 39°32' West [27].

Data were obtained from February to June 2005 by means of open-ended interviews using the pattern techniques of the ethnographical research focused on the cognitive anthropology [28]. An Open and Clarified Consent Term was elaborated based on the National Health Council Resolution number 196/1996, which rules the ethical aspects of the research involving human beings. It was read to the villagers and distributed among those who participated in the study. Thirty people of both genders whose ages ranged from 13 to 86 years old were interviewed. Local inhabitants are mainly small farmers and most of them have an Afro-Brazilian origin. The main objectives of the research were explained clearly in the beginning of each new interview and people were asked if they wanted to participate. The researcher maintained a visual contact with the interviewees using a micro tape-recorder; semi-literal transcriptions are kept at the Laboratory of Ethnobiology of the Universidade Estadual de Feira de Santana (UEFS).

Glass jars filled with alcohol 70% were left in some residencies in order to get some bird-spider species from the area and to make a survey about the collected specimens. Projective tests were possible thanks to these collections, when people were inquired about the content and the common names, the local impressions on the animals, and their uses were noted down. Some specimens of spiders were identified by Dr. Paulo César Motta from the Biology Institute of Universidade de Brasília. They were deposited at the Arachnida Collection: *Lasiodora *cf. *paraybana *(DZUB 3563), Aviculariinae (DZUB 3564) – both Theraphosidae, and *Ctenus *sp. (DZUB 3565), a Ctenidae.

Data were analyzed using the union model, which involves considering all available information on the surveyed subject [29]. Controls were carried out both through consistency checking tests and reply validity tests, which make use of repeated inquiries in synchronic and diachronic conditions, respectively [29]. The former occurred when the same question was asked to different people in very close times; the latter occurred when the same question was asked to the same person in different moments.

## Results and discussion

The generic name 'caranguejeira' designates a myriad of species throughout Brazil (Mygalomorphae spiders, especially those from Theraphosidae and Ctenidae families). Although few kinds of these spiders have been cited during the actual research, none of them were distinguished by specific names. However, they are differentiated from each other based on their body size and color, as well as the locality they come from. The way informants recognize the local 'bird-spiders' is provided below:

"I only know one kind, the large one" (Mrs. Z., 51 years old).

"The true ones live in the dry lands" (Mr. E., 52 years old).

"Those ones from here are smaller and reddish" (Mr. E., 50 years old).

"The bird-spider is like a crab" (Mrs. P., 80 years old).

"The one from the hill [Serra da Jibóia] is very black, but the other is gray" (Mrs. N., 67 years old).

"There are large and small bird-spiders" (Mrs. S., 82 years old).

"In the caatinga [semiarid vegetation] it is large and black. The ones from here are smaller and reddish, a little dark" (Mr. E., 50 years old).

It was observed that all specimens of local bird-spiders are named under the label 'caranguejeira'. Sometimes, however, informants refer to them as 'crab' because they say these hairy spiders resemble such a crustacean due to their general morphological traits. This association is known in other parts of the country [30].

In the ethnozoological classification system of the inhabitants of Pedra Branca, bird-spiders and all other arachnids are perceived and categorized as 'insects'. This can be observed in the following testimonies:

"Insect is a dangerous object, is not it? Scorpion and spider are hazardous" (Mr. Z., 54 years old).

"Insects are those small animals: ant, beetle, spider" (Mrs. E., 84 years old).

"The spider is a bad insect" (Mr., A., 42 years old).

The inclusion of different, not systematically related animals into the ethnozoological domain 'insect' has been verified in the literature. The way human societies construct the ethnocategory 'insect' has been explained through the Entomoprojective Ambivalence Hypothesis: human beings tend to project attitudes and feelings of harmfulness, danger, irritability, repugnance, and disdain toward non-insect animals (e.g., toad, rat, scorpion, spider, lizard, snake, bat, earthworm, among others), by associating them with the culturally defined category 'insect' [31]. The idea of ambivalence comes from sociology and relates to attitudes that oscillate among diverse, and sometimes, antagonistic values. Projection results from the psychological processes by which a person attributes the reasons for his/her own conflict and/or behavior to another being. Nolan and Robbins [32] state that the organization of ethnozoological semantic domains (e.g., 'mammal', 'snake', 'bird', 'fish, 'insect', etc.) is influenced both by the emotive meaning and the culturally constructed attitudes toward these domains. Indeed, the way people perceive, identify, categorize, and classify the natural world changes the way they think, act, and feel in relation to the animals. In other words, folk taxonomies are based not only on the knowledge of biological characteristics (cognitive dimension), but also on feelings (affective dimension, here including the ideological dimension) and behaviors (ethological dimension).

Weather working on folk biological classification systems, the researcher should be aware not to consider cognitive categories as universal. Instead, they have to be 'discovered' through a proper methodological approach that reveals the conceptual paradigms [33]. For instance, in their folk entomological classification system the Kayapó Indians from the Brazilian State of Pará categorize animals with shells and no flesh as equivalent to insects [34]. The Kaxinawá Indians from Alto Juruá, Amazon forest, have the classificatory category *mabu txakabu *(literally, 'useless thing') to designate the different types of animals, such as snakes, centipedes, spiders, and ants. And to the *seringueiros *(rubber tappers), those terrestrial or flying animals that sting and have poison are classified as insects (e.g., snakes, wasps, scorpions, Ponerinae ants, and bird-spiders) [30].

Informants provided some information about where bird-spiders live and can be found, such as termite nests, tree holes, soil holes, rotten logs, litter, fence and wall base, rubbish, abandoned houses, storage rooms, rooftops, banana trees, inside bromeliads, garbage, and piles of things. Literature says that most part of their time these spiders live isolated, generally living in averted places (trees, termite nests, burrows in cliffs, and underground galleries). Big bird-spiders of the subfamily Theraphosinae inhabit under the trunks of rotten trees, near roots, in natural holes, and inside termite nests [3,9]. *Avicularia *spp. live on trees.

According to the interviewed people, bird-spiders are more easily seen during rainy season, especially after thunderstorms when people see them wander alongside the roads or walking on roofs:

"It likes the tiles. When it is thundering it keeps *friviando *[walking on roofs]" (Mrs. D., 70 years old).

"The bird-spider walks when it thunders. It appears more in the summer, in thunder times" (Mrs. P., 80 years old).

"It likes the winter better. When it rains and stops, we see them a lot" (Mr. A., 42 years old).

"When it rains it keeps walking. It goes to the asphalt, to a more open place" (Mr. E., 50 years old).

"It appears in thunder times" (Mr. J., 66 years old).

"Here in the woods it is difficult to see it, but in the caatinga we see this insect whenever there are thunderstorms" (Mrs. V., 66 years old).

Being a nocturnal arthropod, bird-spider only leaves its hideout at night or during cloudy afternoons, after summer thunderstorms when the sky is still covered with clouds, or during daytime in the inner of shadow forests [35]. It may be seen out of its burrow during daylight, although it seldom moves more than fifteen or twenty centimeters from its burrow [11]. It is interesting to notice that in Brazilian Northeastern it is said that when it is going to rain the bird-spider leaves its burrow and starts walking [36]. Maybe this behavior is influenced by the barometrical pressure that increases with the coming winter [37].

In addition, mature males usually leave their burrows to search for and breed with females. In the dryer areas of tropical habitats, rainfall and humidity are presumed to play more profound roles in bird-spiders' annual cycles, determining the season in which eggs are produced and molting occurs [5,11]. According to Schultz and Schultz [11], the presumption is that spiderlings are produced at that time of the year when food and moisture are most plentiful, probably immediately following the rainy season. That is why informants use to see vagabond spiders during wintertime.

In relation to the knowledge people have regarding bird-spider's food habits, the following testimonies were recorded:

"It feeds on beetle, centipede. What it finds it feeds on. It only eats insects" (Mr. A., 42 years old).

"It can be a cricket, a caterpillar, the same spider it takes. It is what it finds" (Mrs. P., 80 years old).

"It lives on small insects, mosquito, theses things" (T., 38 years old).

In nature, bird-spiders will eat almost anything that moves and is small enough to overpower. They have been known to eat small rodents, small lizards and snakes, small birds, insects and spiders, even other bird-spiders [11]. Because bird-spiders prey on scorpions, tarantulas, and armed spiders they must be considered as being useful to man [9]. One has already recommended that these spiders should be protected because they also feed on snakes [35].

Informants also talked about the animals that prey on mygalomorph spiders:

"The *guará *[raccoon] also eats it. Snake feeds on small bird-spiders" (Mr. T., 39 years old).

"It can be a snake. It is a snake" (Mrs. P., 80 years old).

"The wasp feeds on it. What does it do? It goes, digs the soil, then it returns, carries it [the spider], and puts it in the center of the hole. It is stringer than the spider" (Mr. E., 67 years old).

"Only the *cavalo-do-cão *[spider-hunting wasp, Pompilidae] can kill a spider. People tell that" (Mrs. V., 667 years old).

"It [the spider-hunting wasp] sits on it and kills it. It sucks the spider entirely" (Mr. A., 73 years old).

One interviewee reported that once a cat died after eating a bird-spider and then it died. That is why she buries spiders in order to prevent dogs and hens from eating them. Bird-spiders' natural enemies are storks, javalinas, skunks, coatimundis, owls, lizards, snakes, centipedes, scorpions, and spider-hunting wasps (Pompilidae). But the most impressive are huge *Pepsis *wasps called tarantula hawks. As Conniff [7] stresses, "The wasp's bold strategy is to slip directly under the venomous fangs and plant its stinger in the tarantula's soft tissue. The effect on the tarantula is immediate paralysis. The wasp then grads it off to burry as a macabre nursery for its offspring, laying a single glistening white egg on the victim before covering it. When the egg hatches, the wasp larva will dine on the living tarantula, avoiding the vital organs at first so that its immobilized food supply remains fresh for a month or more."

Besides tarantula hawks, small-headed flies (family Acroceridae) also parasitize these large spiders. Acrocerids lay their eggs on the spiders; when the eggs hatch, the larvae enter the body of the spider and consume internal organs [5].

Considering traditional knowledge on bird-spider's reproduction, informants said that:

"The spider makes a ball that is full of small spiders inside. I think it is the same with the bird spider" (Mrs. P., 80 years old).

"In June they are all hatching. All the insects. There is a time it has a ball of eggs. At the rainfall period. I think they are born inside the hole" (Mrs. V., 67 years old).

In fact, females spin a walnut-sized eggsac holding as many as one thousand eggs or more. They tend it carefully, airing it at the burrow's entrance and protecting it from predators [11].

During interviews, it was noticed that the most commented aspect of the interaction between bird-spiders and inhabitants of Pedra Branca is related to their dangerousness. People said that these spiders are very venomous and can cause health problems. Some of the individuals have compared the spiders' venom with that of snakes. Although they have recognized that bird-spiders can bite, they are more frightened with the hairs that cover their back and legs. According to their perception, the venom is in the hairs. Fortunately, nobody has recorded fatal episodes:

"That bites us. It has venom like the snake. If it bites, it is worse than the snake. Even dead I am afraid of that. Even the hairs, if we tread on them, they burn like a fire. We do not facilitate with these insects" (Mrs. P., 80 years old).

"The problem is not the sting, but its hairs. Its venom is in the hair" (Mrs. E., 56 years old).

"The hair broils, burns like fire. It blisters. It is like the venomous caterpillar" (Mr. A.M., 78 years old).

"I am afraid of its sting, but I am more afraid of the hair. We feel sick with fever and diarrhea. It discharges the hair. If you are going to take it, you should do it for the wind. The hair is fragile. When we touch it, it releases. It only presents hairs in its back and in the legs" (L., 28 years old).

In order to alleviate the local symptoms caused by the contact with those hairs, that is, the formation of blisters, people recommended some local healing practices, such as putting garlic, ice, some green leaf, cashew nut, and even gasoline on the affected area.

The Navaho Indians from North America say these spiders are more poisonous than the black widow, that death occurs within half an hour after being bitten unless treatment is given, or even that there is no effective treatment as there is for spider bite. A traditional medicine prescribed for bird-spider bite and scorpion sting was the gall of wolf, bear, mountain lion, bob cat, skunk, and eagle mixed with corn meal and 'peppery plant' [38].

An interesting comment was made on one aspect of the spider's behavior. According to the interviewed individuals, bird-spiders are supposed to run after someone when it gets 'angry': "It gets enraged, thus it runs after us. It is brave. It is lightness! When we tease it, it runs after us" (Mrs. L., 68 years old); "When it gets angry, it puts its legs upwards in anger. It runs after us" (Mrs. V., 67 years old). This behavior is also associated with the reproductive period, as informants said:

"When it is hatching, it gets very angry" (Mrs. E., 86 years old).

"When it is hatching, it runs after us" (A., 25 years old).

"It ran after a brother of mine. Hatching time is when it spawns and gets in a hatching mood like a hen" (Mrs. D., 70 years old).

Bücherl [9] observed that *Acanthoscurria *spp. are fierce spiders; they lift up their bodies in position of attack and some of them can bite with no difficult. *Acanthoscurria gomesiana *Mello-Leitão, 1923, when disturbed, quickly assumes a defensive attitude elevating its front legs and trying to bite [35]. *Lasiodora klugi *(Koch, 1850) usually lifts up in an attack pose and projects against the intruder, or then it turns its abdomen into the direction of the enemy and bombs it with a cloud of fine urticating hairs. And what concerns reproduction and hostile behavior, literature says that females can be quite aggressive while guarding their eggsacs [11]. However, they do not bite unless they are severely provoked [5].

The venominations and accidents caused by those arthropods of the Araneae order are termed araneism, which comes from the Latin word *araneus, aranea = spider*. Bird-spiders cause accidents with small repercussion, and there are no recordings of serious accidents between Mygalomorphae spiders and humans in Brazil [4,39]. Lucas *et al*. [40] recorded 91 cases of bites attributed to mygalomorph spiders at the Hospital Vital Brasil, Instituto Butantan, from 1966 to 1991. Bird-spiders' bites, however, usually just cause a low intensity and short duration pain, sometimes accompanied by a local discrete inflammation [41]. Bites of the spiders of the genus *Atrax*, especially *A. robustus *(Cambridge, 1877) from Australia, are exceptional, producing severe envenoming symptoms [40]. Data confirm that non-aggressive spiders do not harm health since "No case of accidents caused by bird-spiders in humans has been reported in Brazil as the cause of death" [9].

Spiders of the Theraphosidae family (Theraphosinae, Aviculariinae and Grammostolinae subfamilies) present an area covered with hairs that have little urticating bristles on the back of their abdomen, these hairs are visible only through the microscope. This area can contain 10,000 to 20,000 hairs per mm^2 ^[1,3]. When it feels threatened, it rubs its hind legs against its abdomen flying these hairs away. When they hit human skin, these hairs can cause irritation and severe itching [4]. These hairs have also caused lesions on the cornea. They can also reach the high respiratory treat causing severe irritation. Symptoms however can depend on the species as well as on the sensibility of the victim [42]. It is observed local mild pain, and there is little exhuberant erythema and edema. Relief begins after 1–2 hours and no serious accidents in humans have been reported. Treatment consists only in the alleviation against pain. In those cases involving irritative or alergic phenomena, corticosteroids and antihystaminics are administered through systemic way, and corticosteroids through topical use if the lesion is local.

## Conclusion

In general, Pedra Branca inhabitants' traditional zoological knowledge regarding bird-sipders has shown to be rationally coherent with the academic knowledge. The complex set of feelings, knowledge and behavior towards these arachnids and other arthropods as well may be translated as a valuable cultural resource, which in turn has to be taken into account both in the developmental processes addressed to the region – maybe the transformation of it in some kind of Conservation Unity, and in studies of local fauna inventory.
